# An Ethnographic Perspective on Urban Planning in Brazil: Temporality, Diversity and Critical Urban Theory

**DOI:** 10.1111/1468-2427.12765

**Published:** 2019-05-06

**Authors:** Martijn Koster

**Keywords:** urban planning, temporality, diversity, ethnography, critical urban theory, anthropology, Brazil, Recife

## Abstract

This article provides an ethnographic perspective on urban planning by presenting the creative practices of marginalized slum residents in Recife, Northeast Brazil, who are affected by planners’ decisions. It argues that such a perspective contributes to current critical urban theory in three ways. First, while many studies of urban planning follow the temporality of the timeframe of a particular project (‘project time’), this analysis emphasizes the timeframe of the lives of the affected residents (‘people's time’). Second, it attends to diversity, taking account of the variety of affected residents and the diverse consequences of urban planning on their lives. Third, it shows how urban interventions — similar to marriage, divorce, the birth of children and the death of loved ones — are high‐impact life events for the urban poor. Finally, the article assesses the engagement between ethnography and critical urban theory and argues in favour of ‘grounding’ the latter better in the analysis of actual practices and experiences.

## Introduction

In a rapidly urbanizing world, urban planning, including the interventions it produces, increasingly affects many people's everyday lives. As a domain of interventions into urban space and the lives of residents, it has been studied from different angles, but the perspective of the individual city dweller is not often taken into account. This article analyses how urban residents experience and deal with urban planning and how it affects their lives. It seeks to respond to the recent call for employing more ethnography in urban and regional studies and geography (Megoran, 2006; Bunnell and Harris, 2012; Thieme *et al.*, 2017). It also approaches urban planning from the vantage point of marginalized city residents to yield more grounded representations of urban planning and its impact on urbanites.

Across the globe, policymakers largely have an instrumental view urban planning, focusing on the design of interventions into the urban landscape and their presumably benign goals (Scott, 1998; Bueno, 2000; Sanyal, 2005). Critical studies countering this point of view are plentiful, ranging from critiques of how planning governs urbanites’ lives to Marxist analyses of accumulation by dispossession. To complement these critical studies, this article emphasizes the value of an ethnographic approach to the study of urban planning, specifically by highlighting the lives of the residents who are affected by it. An ethnographic approach contributes to the debates on urban planning in three ways. First, it allows scholars to see the multi‐temporality of urban planning, as residents’ temporalities differ from the temporality of planners and their projects (Abram and Weszkalnys, 2013; Miraftab, 2017). Second, it can capture the variety of practices and perspectives of affected residents and the diverse consequences of urban planning on their lives. Finally, ethnographic research can show how urban planning interventions are recurrent and high‐impact events in the lives of marginalized, yet agentive, city residents.

This study is part of a broader research project that consisted of 24 months of ethnographic fieldwork in Recife, Brazil. I started my fieldwork in 2003, conducted most of it between 2004 and 2006, and returned to the field for substantial follow‐up studies in 2009, 2015 and 2018. I lived in one of the low‐income neighbourhoods for 18 months and followed various urban planning interventions. Based on participant observation and formal and informal interviews, I have constructed life histories of several people affected by urban planning. The emphasis in these stories is on what people do: when and how they move to different places, how they face urban interventions, and how they negotiate their space in the city with the authorities and other city residents. Their personal stories present an intimate perspective on wider processes of urban change and their entanglement with individual lives (Long, 2001; Locke and Lloyd‐Sherlock, 2011). Over the years, I systematically returned to the same people to see where and how they were living, gaining a longitudinal view of their lives and their dealings with urban development. The vignettes I present are based on a combination of longitudinal ethnography, in which I studied the same people diachronically over a period of 15 years, and their oral histories.

I first briefly describe different perspectives on urban planning and the debates they have generated in recent years. Second, I position an ethnographic approach within these debates, highlighting how it can contribute to our understanding of urban planning and its effects. Third, I present different narratives of residents, concentrating on issues of temporality and diversity, and on how urban interventions have become recurrent and high‐impact events in people's lives, causing them to renegotiate their space in the city. I conclude with a reflection on the fertile dialogue between ethnography and critical urban theory and, finally, suggest how practitioners can benefit from the insights in this article.

## Different perspectives on urban planning

Policymakers largely hold an instrumental or interventionist perspective on urban planning, seeing it as a domain that deals with the technical, environmental, social and political processes concerned with the use of land and the design of the urban environment, including transportation and communication networks. Urban planning, from this point of view, aims at ensuring a regulated and formalized development and management of urban spaces and populations. As such, it is concerned with the allocation of resources to urban residents, in terms of their housing, living conditions and access to services in the city. In Brazil, especially since the 1960s, urban planning has been carried out in a context of modernity, national progress and development (Holston, 1989).

The instrumental perspective starts from the premise that urban planning solves a problem. Its solutions may vary from the improvement of housing and access to public services to the demolition of informal settlements. Since the 1990s, improving the social conditions of the urban poor has been an important component of the Brazil's highly acclaimed urban reform (Klink and Denaldi, 2016). Residents of slums or other places deemed unsuitable were not to be simply removed, but given substitute housing. The last two decades have witnessed a shift from top‐down modes of urban planning towards more participatory approaches. City administrations have used such approaches as a means of deepening democracy, centring upon citizen representation and the equal distribution of resources among city residents (Fainstein, 2000; Fung and Wright, 2003). The internationally acclaimed participatory budgeting programmes in Porto Alegre and Recife have been models for other cities both in and outside Brazil (Baiocchi and Ganuza, 2014; Caldeira and Holston, 2015). Across the globe, such participatory forms of planning are often framed along notions of the democratic city and social inclusion. More generally, urban planning projects tend to be carried out in the name of beauty, progress, health, sustainability and accessibility (Ghertner, 2011; Harms, 2012).

Critical studies of urban planning, in contrast, criticize this instrumental approach because it is ‘narrowly focused on master plans and the instruments of land‐use planning’ (Klink and Denaldi, 2016: 414) and it leaves little space for residents’ participation (Cunha, 2018). Such studies show how urban planning (re)produces inequality among residents because of citizens’ inadequate access to governance and the imbalanced allocation of resources (Harvey, 2003; Miraftab, 2009). This perspective presents an often‐dystopian narrative in which urban planning serves the needs and aspirations of upper class, by redesigning the city and facilitating gentrification, urban segregation and the building of business districts (Vargas, 2005). Furthermore, urban planning can be used to ‘purify’ certain spaces in the city (Appadurai, 2000; Olds *et al.*, 2002). In an anthropomorphic conception of the city, urban planning ‘cures’ or removes the infected elements of the urban body. Valladares (2000: 15), in a historic overview of urban policies regarding *favelas* (slums) in Rio de Janeiro, demonstrates that these marginalized neighbourhoods were long considered a ‘leprosy to aesthetics’.

Recent critical studies approach urban planning from a ‘governmentality’ (Foucault, 1991) perspective, arguing that states discipline populations to behave in desired ways through techniques such as spatial planning and citizen participation (Ong and Zhang, 2008; Blakeley, 2010; Rutland, 2015). Residents may disagree with urban interventions, yet eventually they are made compliant, disciplined by institutional structures and dominant discourses (McGuirk and Dowling, 2011; Huisman, 2014). Scholars adopting a governmentality perspective often concentrate on discursive formations, analysing policy documents and the formal manifestations and procedures of planning.

Other critical studies highlight how the neoliberal capitalist policies that drive urban planning across the globe result in a centralization of wealth and power in the hands of a few by dispossessing the rest of the population of their wealth or land (Harvey, 2003). Studies of ‘accumulation by dispossession’ demonstrate how urban planning violates many urbanites’ right to the city by excluding them from a life in the city (Lefebvre, 1991; Banerjee‐Guha, 2010; Souza, 2018). Sometimes the exclusion takes a very direct form, as in evictions. Often it occurs indirectly through security measures and gentrification, explicitly or implicitly spurred by moral understandings of who is considered an asset to the city (de Koning, 2015). Several studies demonstrate how social movements and residents’ initiatives may challenge the exclusionary workings of planning, with different results (Shin, 2013; Lelandais, 2014). In Recife, the Ocupe Estelita movement, inspired by the Occupy Wall Street protests, gained international attention, especially when the renowned Marxist scholar David Harvey visited and gave a speech (Harvey, 2014). When historic warehouses at the waterfront were being replaced by high rises, activists occupied this area and protested the ‘separation of capitalist development and democracy’ (Pìnto, 2014: 1; Nóbrega, 2016).

Building on and advancing these critical insights, this article discusses the contributions of an ethnographic approach to the study of urban planning. Ethnography, originally developed in anthropology and with a long history in many disciplines across the social sciences, focuses on people's lived experiences. It entails an effort to understand people's practices and the ways they give meaning to these practices, in their context of acting and thinking (Stoller, 1989; van Hulst *et al.*, 2015). Its main method is participant observation, which requires that the researcher is present in the field. In other words, the ethnographic approach ‘entails a commitment to trying to understand the world from another's perspective through sustained engagement in their social environments and participation in the practices that render those environments meaningful’ (Reeves, 2011: 907). Focusing on residents from low‐income neighbourhoods in Recife who are confronted with urban planning, my methodology specifically builds upon the ‘actor‐oriented approach’. This ethnographic approach is employed in development sociology, to analyse residents’ practices and perspectives around particular interventions (Long, 2001; Nuijten, 2003). It concentrates on how marginalized people cooperate in, protest against, and modify such interventions. While taking into account structural changes and constraints, an actor‐oriented approach starts its analysis with the creative agency of local actors. Agency, in this sense, is always situated: it exists in relation to the broader context, meaning that people's practices are involved in and constrained by wider developments and forms of governmentality, and that they may work to intervene in and challenge such larger structures of rule (Merlan, 2016).

In contrast to critical studies that centre on a singular rationality of disciplinary governance or the impact of political economic processes, my study looks at the interplay between the power relations inherent in urban planning and how these govern human conduct, on the one hand, and residents’ creativity and their room to manoeuvre, on the other (Anjaria, 2016). It acknowledges that governmental attempts at discipline are always incomplete—fragmented in light of the resistances, refusals and negotiations of individual actors (Clarke, 2004; Sletto and Nygren, 2015). This perspective foregrounds residents’ *mētis*, the wide range of practical skills and know‐how that they use for navigating the constantly changing urban environment (Scott, 1998). In so doing, it analyses how people make sense of and navigate the city through (re)appropriating space and creatively dealing with the interventions they face (Anjaria and McFarlane, 2011).

## Contributions of an ethnographic approach

An ethnographic approach contributes to our understanding of urban planning in three ways. First, with regard to temporality, it allows us to perceive that ‘people's time’ is different from ‘project time’. Recent ethnographic studies discuss the multiple temporalities involved in urban planning (Abram and Weszkalnys, 2013; Caldeira, 2017). They point to a diversity of ways to remember the past and imagine the future (Cavalcanti, 2009). In contrast, other studies share an important shortcoming: their analysis unfolds along the temporality of the intervention, the ‘linear, progress‐oriented temporality of planning’ (Harms, 2013: 349). In this framework, residents are identified as a ‘target population’ and their identities are constructed in relation to the intervention. In following the timeframe of the project, such analyses leave all before and after the intervention as ‘empty time’, time in which nothing happens or exists (Raco *et al.*, 2008). Ethnography, instead, focuses on people's time: the temporalities of the affected residents. By foregrounding the affected residents’ livelihoods and personal stories about the past, present and future, ethnographic research can document how localities are socially configured prior to interventions and how residents continue to work towards multiple futures after the planners have left (Nielsen, 2011; Miraftab, 2017).^1^

Waiting and uncertainty are often central in people's temporal experience of urban planning. Like the anthropologist Eric Harms (2013: 346) does in his study of urban planning and forced eviction in Ho Chi Minh City, I show ‘how people in eviction zones must cope with and also take advantage of unfamiliar and largely alienating temporal relations marked by uncertainty, ambiguity, and contradiction’. While the timeframe of an intervention may be clearly formulated by policymakers and project developers, such projects often involve long periods of waiting and uncertainty for the residents involved.^2^ Indeed, residents are often left to wonder: Will my house be demolished? If so, when will this happen? And then, where will I go? Across the globe, as other studies show, urban planning instils uncertainty about the future in the lives of the people affected (Datta, 2016; Waldorff, 2016).

Second, an ethnographic approach is able to capture the diversity of the affected population. People have diverse livelihoods and family situations, express divergent needs and aspirations, and live in heterogeneous urban landscapes. They have different feelings of belonging and different levels of participation in projects and programmes. Some are tenants, others are squatters, while still others have property rights. An ethnographic study of residents’ practices and experiences would pay attention to how urban planning produces new subjectivities, as it may disperse populations and influence their social, economic and political configurations (Doshi, 2013). In so doing, it empirically criticizes a monolithic view of residents as ‘beneficiaries’ or ‘victims’. Harms (2013), in his study of urban planning and eviction in Saigon, for example, shows how two groups of residents dealt with the same intervention in different ways. Ethnographic studies can trace how residents creatively rework urban development projects, their individual preferences related to housing, and their formal or informal arrangements to safeguard their place in the city (Andersen *et al.*, 2015; Koster and Nuijten, 2016).

Third, approaching urban planning ethnographically can provide us with empirically rich accounts of how such interventions have become an incessant presence in people's lives, with an impact similar to major life events. Several studies have demonstrated how urban interventions in Brazil have structured the lives of the urban poor, just like major life events such as marriage, the birth of children, divorce, migration, and the death of loved ones (de Jesús, 1963; Perlman, 1976; 2010). Even when urban interventions do not result in displacement, as Eugênia Motta (2014: 148) shows in an ethnographic study of housing in a Rio de Janeiro *favela*, people's life stories are structured along the transformation of their houses in such a way that it becomes a supplement to, as she phrases it, people's ‘temporal landmarks of births and deaths’.

## Dealing with urban planning: different stories

### Recife, its urban planning and its marginalized residents

This section presents people's life stories, which demonstrate how urban planning interventions are part of life in the city. It includes stories of six different residents and their families, all living in a marginalized urban area in the north of Recife. Recife, with an estimated 1.6 million residents in its municipality and 3.7 million residents in the metropolitan region, has traditionally been the destination of large inflows of migrants from the arid rural areas of north‐eastern Brazil. Many of these migrants have settled in illegal dwellings and, over the years, have lived through several urban interventions that targeted their homes and surroundings. Currently, official numbers state that 23% of Recife's total population lives in irregular settlements or, as they are called, ‘*aglomerados subnormais*’ (subnormal agglomerations) (IBGE, 2010; Nadalin *et al.*, 2014).^3^ Recife's urban reform has been highly acclaimed because of its pro‐poor approach, which has emphasized social inclusion and presented urban development as a vehicle to improve the living conditions of the poor, especially through its Plano de Regularização das Zonas Especiais de Interesse Social (PREZEIS; Regulatory Plan of Special Zones of Social Interest), a system of laws that was implemented in 1987 to legalize the slums and provide them with infrastructure (de Souza, 2001; Leite, 2007; de Vries, 2016). An important dimension of PREZEIS is that it prioritizes shelter and user rights over property rights. Furthermore, through building regulations, it sets out to avoid land speculation. Through PREZEIS, several poor areas in the city have become recognized as protected areas.

In the event of eviction, authorities in Recife and many other Brazilian cities had long provided residents with substitute housing, in contrast to many other countries in Latin America (or elsewhere) where eviction is tantamount to expulsion from the area. However, in the last decade, this pro‐poor ethos has changed, as manifested in the developments for the 2014 World Cup and the 2016 Summer Olympics, in which commercial interests trumped social ones (Santos *et al.*, 2014; de Souza, 2017; Souza, 2018). Various studies demonstrate that urban planning in Brazil has, recently, been replaced by real estate interventions (Rolnik, 2013; Nobre, 2017). The residents in the stories below all migrated to Recife from the countryside. Although each of their stories is different, they together demonstrate how urban planning interventions are important and recurring events in their lives.^4^

### Ana and Bina: two neighbours’ divergent paths

Ana and Bina were long‐time neighbours and friends.^5^ Their stories demonstrate the multi‐temporality of urban planning, the heterogeneity of a ‘target population’ and the different impacts the interventions have on their lives. When I first met Ana in 2003, she was living in the Jacarezinho, an extremely marginalized settlement that was built along and over a brook that had turned into an open sewer. In 1992, Ana had moved to Recife from her birth town in the countryside, occupied a piece of land, and built a shack in the Jacarezinho settlement. When I first met her, Ana lived with her son and daughter, then 11 and 9 years old, in a small shack made of wood and scrap material. The room could barely accommodate one double bed, which she shared with her children. Half of the hut, located just above the water surface, was built on stilts in the bed of the brook. When it rained, the water entered the shack, flooding it a few inches deep. On sunny mornings after rainy nights, Ana and her neighbours put their mattresses out to dry on the street.

In 2003, the Jacarezinho area was selected as a pilot in the Prometrópole urbanization programme, making its residents part of the ‘target population’. Prometrópole was an infrastructure programme in low‐income areas along the Beberibe River, which divides Recife from its neighbouring city Olinda to the north. According to official documents, it aimed at improving the infrastructure of the city, including sewage and drainage systems and the construction of roads. After many years of negotiating, the World Bank, the state government of Pernambuco, and the neighbouring municipalities of Recife and Olinda signed a contract that signified the project's official start (World Bank, 2003). Part of the project involved the demolition of shacks along the riversides and the resettlement of those residents to new housing estates close to the original area. In this way, it was argued, the programme would simultaneously improve the built environment, restore the quality of the natural environment, and reduce poverty. Jacarezinho, as a pilot site, would be among the first areas where demolition and resettlement would take place.

The plans instilled much uncertainty in Ana, which caused her to worry. For a long time, she did not know when the project would be carried out, if she would be compensated for the loss of her house, or if the compensation would take the form of money or a substitute house. Many residents opposed the plans, as they were afraid of losing their homes and livelihoods. One neighbour said during a meeting about the plans: ‘I have a street‐level front window shop. What will I do if I have to move to an apartment on the first floor?’ Ana, although at times afraid, was also hopeful. She told me how she dreamt about a new house and no more flooding. One of her neighbours said, full of hope: ‘The *favela* will turn into a city’, meaning a place with asphalt roads and basic services, where one could live a decent life. In 2003, after the World Bank and the authorities signed the contract, a survey was carried out. Surveyors came and included Ana's house in the register.

Bina, who lived next to Ana, was also included in the survey. In 2001, she had moved into her husband's shack in the Jacarezinho, together with her son. Before that, she had lived in different place in the city. Like Ana, she made a living by washing clothes and cleaning houses for families in the area surrounding the *favela*. Her husband, Naldo, was a handyman who repaired everything from bicycles to fans. Bina also longed for a new house: ‘That is my dream. To have a house of my own. I would really like that’. Both Ana and Bina had to wait for many years before Prometrópole was actually carried out. During this time, the municipality organized many meetings to inform the residents about the programme's progress. Ana always participated, while Bina went only occasionally. As they had been waiting a long time, and the floods recurred frequently, a group of residents organized a protest, burning tyres on the neighbourhood's main road. Bina participated but Ana did not, because she ‘d[id] not like that kind of thing’. The protest attracted the attention of journalists and was broadcast on television.

Four years after the contract was signed, in March 2007, their shacks were torn down. Ana and Bina received a monthly financial compensation (called ‘housing aid’ or *auxílio moradia*) of BRL 151 (then US $60). Ana then rented a place nearby. Bina and Naldo rented a shack on one of the hillsides where Bina had lived before. She explained that the rents were low, and since she already knew many people there, she and Naldo hoped to be able to find some work. However, as the housing aid was barely enough to rent a room, they ended up in shabby places where they did not want to stay. ‘One place had rats’, Bina said. Within a year, she and Naldo lived in three different places, until they finally, like Ana, in April 2008, moved into their new house in Portelinha, a resettlement estate located 500 metres from the Jacarezinho. Bina and Naldo, who had a heart condition, had received a specially designed house for elderly people, with a bedroom on the ground floor. Ana had received a regular house, with two bedrooms on the first floor.

Both women expressed their contentment with the houses and with the location, as they remained close to the families they were working for. They were very happy that they were ‘lifted out of the mud’. Bina said: ‘Here, there is no more flooding, no more cockroaches and no more rats’. Ana was in high spirits to have ‘a real house of my own’. But they did not like that they were no longer next‐door neighbours; in the relocation scheme, the residents had been spread out over the new estate.

In 2009, when I met Ana and Bina again, they still lived in Portelinha. Many other people on the estate had sold their houses. Prices were low at first because there had been a lot of violence when drug gangs were trying to extend their territory to the new streets. Eventually, the violence decreased when a new balance of forces was reached, including more police patrols. As a result, prices went up and it became more attractive to sell. When I returned again in 2015, I found that Bina had sold the house. Naldo had passed away and Bina had needed the money. She said: ‘I had the house, but I did not have anything else. I wanted to move ahead, but I did not have the means to do so’. So she sold the house and bought a shack for her and her son on the other side of the Beberibe River. With the money that remained, she bought furniture and home appliances.

Meanwhile, Ana and her two children, now 23 and 21 years old, still lived in the same house, along with her daughter's two‐year‐old child. Theirs was one of the few houses that had not been altered from its original design. Most residents had, over the years, ‘pushed’ their façades forward, appropriating part of the pavement, or covered their backyards to create more living space. Some had even built an extra floor. Ana had not been able to afford this. There was not much work in washing and cleaning, and the economic crisis, which started in 2015 and was coupled with a political crisis that resulted in the impeachment of president Dilma Rousseff in 2016, had only worsened the situation. In addition, her new job as a door‐to‐door cosmetic salesperson did not provide her with a regular income. Her son married in 2016 and moved to another neighbourhood, to a house that belonged to his in‐laws. Not long thereafter, her daughter married and moved out as well. Both children kept on contributing to Ana's household as much as they could.

### Beto: moving from place to place

Like many poor city residents his age, Beto, born in 1966, had migrated to the city from the countryside. Beto's story demonstrates how people's time differs from project time, and how people may be impacted differently by urban planning. Most of all, his story is an example of how urban planning can be a recurrent and high‐impact event in the lives of marginalized city residents, spurring them to negotiate and re‐appropriate a place in the city. Urban interventions, his story makes clear, are life events, just like marriage, the birth of children, divorce, migration, and the death of loved ones, after which people have to try to ‘move on’.

When he was a teenager, Beto moved to Recife to look for work. He ended up in a shack on the banks of the Beberibe River. At the time, many migrants settled on the riverbanks. These settlements disrupted the river flow and caused floods in surrounding slums and middle‐class neighbourhoods. Until the 1980s, attempts to remove the riverbank slums by the then military government were met with stubborn resistance. After a long period of struggle and negotiation, in which slum dwellers, leftist politicians, activists, and liberation theology clergy participated, the government conceded to demands for a resettlement programme close to the river. In 1981, the government allocated houses to the first group of those expropriated from the riverbank in the new neighbourhood of Chão de Estrelas, built on a former coconut plantation. Relocations like this frequently occurred in Recife in this period, in an attempt by the military government to enhance its power base (Assies, 1991). Beto was relocated to Chão de Estrelas in 1987 and 1988, under the first democratic regime after military rule. In this resettlement scheme, Beto was granted a plot, a structure with walls and a roof, and building materials to finish the house. Soon, he started a relationship with his neighbour's daughter, Raquel, who moved in with him. In Chão de Estrelas, none of the residents had a land title, but the PREZEIS secured user rights. Over the following years, large parts of the area obtained sanitation, drainage, electrification and paved roads.

In Recife, Beto had first been self‐employed as a *tapioca* (cassava root) dough vendor. After that, he found work in a Coca‐Cola warehouse close to the Beberibe River. Finally, he found employment in the canteen of a slaughterhouse, which was one of the main employers in the area at the time. There he had an official work card, which guaranteed, among other things, the right to social security benefits in case of illness, disability or imprisonment. Since 2001, after years of working in the slaughterhouse, Beto made use of this right and received an allowance. He had been declared unfit to work due to mental health problems after one of his brothers had been shot dead in a fight. Aside from his social security allowance, Beto earned an income as a self‐employed garbage collector, fetching construction debris and bringing it to a scrap yard with his handcart.

In 2009, Raquel left him for another man. Beto sold the house, left Chão de Estrelas and bought a shack on the riverbank again. Before too long, a new partner moved in with him. In 2012, another urban development programme was carried out, a follow‐up to the programme that had targeted the Jacarezinho.^6^ The plan was to build a new road next to the Beberibe River, to improve accessibility between Recife and Olinda and to stop people from building structures along the riverbanks again. Beto had to move out and his shack was destroyed. While many of his neighbours had to wait for years to receive a new dwelling, Beto was among the first to be given an apartment in a new building in an adjacent neighbourhood, 1.5 km from the original site.

However, Beto could not get used to living in the apartment building. ‘An apartment is no place for me’, he said, ‘I was raised in the mud, but at least it was a place where everybody knew each other’. After some time, he decided to move again. In 2015, he and his partner sold their apartment and rented a house in Portelinha, close to Ana's. The proprietor, a woman from the Jacarezinho, could not get used to the violence there and decided to move ‘to a calmer neighbourhood’. Beto told me that he was saving up to buy a house in Portelinha, also using the money from the sale of his apartment. However, in 2016, when the landlady was selling the house, he had spent most of his money. He and his partner, with their new‐born baby, moved and rented another house across the street.

### Zezinho: moving from shack to house to office

Zezinho was born in 1960, the son of peasants living in a small village around 60 km from Recife. His story, like Beto's, shows how urban interventions are important life events. More generally, his story demonstrates how people can creatively (re‐)appropriate their space in the city in the face of urban planning. In 1978, Zezinho migrated to Recife, dreaming of employment and a better life. Despite not being able to find a job, like so many others, he decided to stay in the city. When I told Zezinho that I wanted to record his life history, he responded very seriously: ‘The story of my life, about how I am today. I am grateful towards God. I did not have anything, but now…’ After a short hesitation he continued: ‘Well, I still do not have anything, but I thank God for the life that I have’.

In Recife, Zezinho lived in a settlement along the Beberibe River, the same area where Beto lived. Here Zezinho met Marta and they had four sons together. In 1985, they were relocated to Chão de Estrelas, two years before Beto would move to the new neighbourhood. There, Zezinho gradually became known as a community leader. In 1991, he founded a new organization, the *União dos Moradores* (Residents’ Union). This organization had its office in a room attached to Zezinho's house. There, he discussed local urban planning projects with residents. He participated in many meetings organized by the municipality about particular interventions and visited officials and representatives of the project developers and construction companies in their offices.

Zezinho's livelihood was rather complex. Apart from working for one year as a cleaner in a hospital, he had never had a regular job. Between 1996 and 2003, he had been self‐employed, selling bread, candy, soft drinks, beer and *cachaça* (sugarcane liquor) from a small shop at home, through his front window that looked onto the street. When occasionally asked to state his occupation on forms, he put down ‘*comerciante*’, a trader or shop‐owner, and told me: ‘This may mean many things. I am a *comerciante*, but lately I am a little out of business’. Otherwise, Zezinho generated revenue through activities related to being a community leader, varying from being given free public transport to attend meetings to rewards in kind for coordinating projects like the distribution of groceries and goods. His office was the central place for these activities, as it was here that he organized meetings and distributed resources. Also, he gained an income through his involvement in electoral politics — something that was very common for community leaders (Koster, 2012). Most of the time, Zezinho worked for a state deputy or a city councillor, receiving a small salary as a campaigner for the party. His wife, Marta, had a job as a domestic worker with a middle‐class family, working two days a week. Their sons had temporary jobs, as janitors, office boys or cleaners, which Zezinho arranged for them through his political contacts.

In 2009, when I visited Zezinho and Marta, they were divorced, living in the same house but sleeping in different bedrooms. Zezinho's late‐night partying and drinking had put stress on their relationship. When I returned in 2015, Zezinho had a new partner, Claudia, and was actually living in the office of the Resident's Union, which Zezinho had closed off from his former house and to which he had added a kitchen. Next to the desks, chairs and filing cabinet, a mattress leaned against the wall and clothes were hanging on a line.

Before moving in with Zezinho, Claudia had lived in a small shack with her daughter along the Beberibe. In 2012, when the area was targeted by an urban development project—the same project that relocated Beto the second time—her shack was demolished. Unlike Beto, she still had not received substitute housing. Every month, Claudia and Zezinho added the housing stipend of BRL 200 (US $55 in 2018) to their joint income, while living in the office of Zezinho's organization. They did not expect to receive a house anymore, they said. Instead, they hoped that the allowance would continue for many more years.

### Maria, Luiz and their neighbours at the square

Like Ana and Bina, Maria had been relocated from the Jacarezinho to a new house on the central square of the new estate of Portelinha, a location that gave her husband Luiz an opportunity to add a bit to his income, working as a garbage collector. Maria's story emphasizes the diversity of residents and their creative reworking of the interventions they face. In 2015, Maria told me that many of her neighbours had already left. Officially, selling the houses was prohibited, but people did it anyway, like Bina had. According to Maria, since the inauguration of the estate in 2008, the majority of the residents had sold their houses. The prices of houses on her block had been relatively high; the place was attractive as it was next to the square and further away from one particular street where loud parties, gunfights and drug traffic took place. Maria pointed, one by one, at the houses on her street and said: ‘That one sold the house, sold, sold, that one swapped houses to move to another place’. Some were renting out their houses while living elsewhere. Maria told me how, shortly after the inauguration, people had first sold the homes’ metal staircases and toilets, and finally the whole houses, as they needed the money more than the house.

Maria and Luiz had stayed. She was happy with the move. ‘There, the water flowed into the house when it rained. We had rats and snakes. Here it is much better’. In the square next to her house, people sat down to chat, eat, drink and organize parties. On one side, a group of men repaired cars in a makeshift outdoor garage. Luiz found their new location attractive with regard to his livelihood as a garbage collector. He used a small part of the square to store recyclable products like iron, aluminium and plastic. By collecting these things, he kept the square and its surroundings tidy. Maria told me that municipal administrators had acknowledged the importance of his work and had promised to build a more permanent depot, but they had never delivered.

The square's initial design had included lampposts. However, Maria said, they had never been used because people had stolen the light bulbs. ‘Between the posts were many black plastic wiring pipes’, she said, and continued with a smile, ‘A neighbour dug them up and sold them. The people from the municipality got very angry’. Another alteration of the original design concerned a big building next to the square that was meant to become a community ballroom, for public events. However, during the project's implementation, somebody bought it and opened a supermarket in it. The owner rented out apartments on the top floor. Maria told me how more people were buying houses and renting out rooms, and argued that while they were not supposed to do so, she could understand it as it generated a good profit for the owners.

### Simone: unfortunate in marriage and in urban planning

The final story is Simone's. Her story demonstrates yet another kind of impact of urban planning on the lives of residents. It also demonstrates, like Zezinho's and Beto's stories, how urban planning becomes interwoven with other major life events. Simone lived with her then husband Edson in a shack close to the Beberibe River; they lived there with their daughter and the two daughters she had from previous relationships. When their marriage did not work out, they decided to divorce. They divided their shack into two parts, one for Edson and one for Simone. Eventually, Simone decided to move to another place with the children. ‘It was too dangerous for the children, so close to the river’, she said, ‘a neighbour's child almost drowned’. Soon after she moved, in 2012, an urban development project started—the same project that made Beto move out of his shack on the riverbank. The programme started with a survey, registering the names of the proprietors. All those registered would qualify for substitute housing. When the surveyors came, Simone's ex‐husband registered her part of the shack in the name of his son‐in‐law, the spouse of his daughter from a previous marriage. In 2014, when new houses were assigned in a neighbouring area, Simone was very cross that her husband's son‐in‐law received one. ‘He never even lived in the old house’, she said angrily.

Simone argued that she was faced with a double dishonesty. First, her ex‐husband's daughter and son‐in‐law received an apartment that should have been hers. Second, her ex‐husband sold his house directly after receiving it. According to Simone, their daughter should have received part of the revenues of the house. She said: ‘She is our daughter after all, who has lived part of her life with her father in the shack’. In an attempt to fight back, she filed a complaint with a social worker working on the project. However, the social worker told her that it was impossible to change the name of the proprietor in the register.

## Understanding urban planning: temporality, diversity, life events

These stories contribute to our understanding of urban planning by shedding light on the timeframe in which people experience and deal with urban planning (people's time), on their diverse identities and circumstances, and on the decisive and recurrent influence of urban planning on their lives. First, looking at the stories of these residents, we see how their experience of urban planning has a temporal dimension that differs from that of urban development projects. In general, projects start with the design process, with signing a contract, followed by a survey. Several meetings are organized to consult the population. In Brazil, if relocation is part of the intervention, the authorities negotiate—often at length—with the owners of the land where they intend to build substitute dwellings. Then they demolish the shacks, provide housing aid for temporary housing, and start the construction of the new housing. The timeframe of urban development projects continues until the completion of the new houses and their official inauguration, often dressed up as a grand ceremony with political bigwigs and journalists.

Following the actors, we see a different kind of temporality. People like Ana, Beto and Zezinho are born in the countryside, move to the city in search of work, and buy or build a shack in a *favela*, often on a riverbank. They organize their livelihoods, build up a network in the neighbourhood, have partners and children, establish a home, and dream about the future. Then an urban planning project is announced. The waiting starts, accompanied by uncertainty. When will they have to move? To where? Will they get financial compensation or a substitute dwelling? Will they have good neighbours in the new place? Will there be employment opportunities there? Eventually, the project expels them from their homes. In Recife, this often—but certainly not always—means that they receive compensation or a substitute dwelling.^7^ However, they first have to spend time in temporary housing. Eventually, people move to their new houses and start to organize their lives again, modify their houses and continue to dream about the future. People may sell their house again, or rent it out to others, due to changing financial conditions or family situations. Often, they move to places where they are once again confronted with urban interventions.

The stories demonstrate the multi‐temporality of urban planning. Although the dominant perspective is that the time before and after the project is not important for analysis, ‘empty time’, these stories show the opposite. Before the formulation of planning interventions, people lived in the area and made use of it. They had full lives there, with social configurations based on kinship, friendship and work. After the project time of urban planning ended, people's lives went on. Most will be confronted with upheavals from urban planning again, perhaps several times more, during their lives. From the perspective of the affected people, the temporal scope of urban development spans from the moment they move into the city (or are born in it) to the moment they die. For them, urban planning interventions are not one‐time things but recurrent events in their lives.

Second, the vignettes show us the diversity of the residents and the impacts of urban planning on their lives. They are not a homogeneous group of ‘beneficiaries’ or ‘victims’. They have different livelihoods, different aspirations and different family situations, resulting in highly diverse ways of dealing with the interventions. Beto, for instance, wanted to live in Portelinha, while the woman who rented him the house desperately wanted to move out. The stories demonstrate that some residents made creative use of the urban interventions, while others were victimized by them. Urban planning brought benefits to some, problems to others, and often combinations of both to most people. Some residents made strategic use of the urban development projects: Simone's husband had astutely, if falsely, registered part of the shack in the name of his son‐in‐law, thereby securing a house for his daughter and her husband. For Zezinho and Claudia, the monetary housing aid was a welcome addition to their otherwise very low income. The owner of the supermarket close to Maria had, like many other more affluent residents, bought several houses and rented these out, making a profitable business. Urban development did not bring the future hoped for by others, like Simone, who ended up frustrated and without any benefits. Although the stories of Ana and Bina are similar, the two friends and neighbours ended up in different situations after the intervention of urban planning in their lives. Ana, even without much income, was still living in her concrete house in Portelinha, while Bina sold her house for reasons of necessity and moved back to a shack.

Third, the different stories show how urban planning is a high‐impact and intrinsic element in the lives of the urban poor. Like their migration to the city, the birth of their children, marriage, divorce and the death of loved ones, urban interventions are important life events for these marginalized city residents. Although cities always change and all urbanites have to deal with change, for the urban poor, such changes are often more uncertain and more intrusive. Their homes are demolished, their networks altered, their livelihoods disrupted. Spatial stability, living in a place with good prospects of staying there, has become a privilege in the continuously changing city—a privilege that is not granted to the urban poor. Instead, they actively work to claim their place in the city and adapt to the dynamics of their living environment. They navigate urban change, making their way through contexts of deep political, economic, social and spatial inequality (Vigh, 2009).

## In conclusion: reflections on the dialogue between ethnography and critical urban theory

This article illustrates how an ethnographic perspective may contribute to our understanding of urban planning. This last section takes account of the dialogue between ethnography and critical urban studies. Ethnography, I argue, extends the engagement of critical urban studies and provides it with the opportunity to ‘ground’ theory even better in people's practices and experiences. This grounded attention to the lived reality of marginalized city dwellers, their practices, perspectives and aspirations, produces an analysis that challenges the dominant instrumental view of urban planning. It shows that people's practices partly align with urban planning projects, while at the same time, those practices partly counter planning logics and goals.

Critical urban studies often tend to rely on a rather unilineal — and dystopian — temporality, that of disciplining rationalities of neoliberal governance or of political economic processes of accumulation by dispossession (Harvey, 2003). Countering such singular uninterrupted timelines, an ethnographic approach emphasizes the presence of different, even contesting, temporalities. Likewise, while the dominant framework in critical urban studies has a tendency to portray ‘the urban poor’ as a rather homogeneous group (Pasternak, 2002), this article shows how an ethnographic analysis foregrounds the diversity of city residents and their different strategies of (re‐)appropriating their place in the city and dealing with change. In so doing, as Jonathan Anjaria (2016: 7) argues, ethnography sets out to capture political economic processes on the one hand and people's personal practices and experiences on the other, illuminating the ‘generative tension’ of the two ‘that is constitutive of urban life’.

This article also shows how individual residents often embrace parts of the dominant planning discourse—they argue that particular interventions make the city more beautiful or that certain projects have improved their lives—y*et al*so how they give expression to counter discourses, resistances and a creative reworking of policies and programmes. Their counter discourses are often full of uncertainty: Will I go? When will I go? Where will I go? Will I be able to make a living there? Who will be my neighbours? This uncertainty seems to be a common way for residents to experience urban planning across the globe (Datta, 2016; Waldorff, 2016).

Sometimes, residents’ practices include acts of resistance—like the burning of tyres in which Bina and her neighbours participated, or the protests we see in many cities elsewhere (Shin, 2013; Lelandais, 2014). An ethnographic analysis takes account of the diversity of the residents and the range of their practices, small and mundane as these may be. Paying attention to the diversity of actors and their diverging needs and aspirations, it can generate a nuanced understanding of how people's lives are entangled with larger urban structures and changes. This perspective does not discard macro‐analyses and structural explanations of such larger changes. Instead, it involves them in a dialogue with more complex and differentiated local understandings. Indeed, as Sapana Doshi (2013: 862) argues, ‘radical scholarship must pay more attention to the often‐overlooked, differentiated embodiments, meanings, and experiences of urban change that are at the heart of such deep social transformations’. Understanding people's local, situated practices is crucial for comprehending wider urban changes and socio‐political transformations.

Urban planning projects, time and again, affect the place of the urban poor in the city—be it directly, as a consequence of eviction, or indirectly, as a consequence of gentrification or other more subtle forms of changing the urban landscape. Under the banner of social inclusion and development, these projects often end up accumulating wealth for those in the richest brackets of the population. It is of vital importance that we continue to question and criticize urban planning through critical theory. Yet, this critical theory must not become detached from actual practices. An ethnographic perspective can contribute to ‘grounding’ critical analyses in the lived realities of the people affected by these developments.

Similarly, for practitioners, understanding the multi‐temporality and heterogeneity of the residents can help them formulate plans and policies that take people's life histories, needs and aspirations more seriously. The timeframe of an intervention may seem linear and progress‐oriented, but for the affected residents it often involves uncertainties and setbacks. Given that the urban poor already face many uncertainties, practitioners should provide clarity regarding, for instance, the dates for relocation, the guarantee of decent substitute housing close to the original location and, if residents wish, with the same neighbours, to maintain their social networks and work opportunities. Also, it would provide useful insights if practitioners revisited resettlement schemes in the years after the intervention to see how, when the planning project is over, people's lives continue in diverse ways.

## Footnotes

1 The need to show a timeframe that is different from the urban planners’ point of view underscores the need for longitudinal studies of affected neighbourhoods (Gohn, 2010).

2 In his ethnography *Patients of the State*, Javier Auyero (2012) demonstrates that, for the urban poor, waiting is perhaps the most characteristic practice of their interaction with the authorities.

3 Juridical differences exist between *favelas* that are the result of land occupations, and clandestine or irregular allotments (*loteamentos clandestinos* or *irregulares*, respectively), that are dwellings that do not (clandestine) or do not completely (irregular) meet the juridical and administrative requirements (IPEA/CPGUR *et al.*, 2002). In practice, these categories are hard to distinguish (do Lago, 2003).

4 These residents, their neighbourhoods and the interventions they faced should not be considered representative of the low‐income neighbourhoods in Recife or in Brazil. It is precisely their diversity that demonstrates the value of ethnography for critical urban studies.

5 All names are pseudonyms, except for one informant who asked me to use their real name.

6 This programme was paid with funding from the federal Growth Acceleration Program (PAC; *Programa de Aceleração do Crescimento*).

7 This research took place under the Recife municipal administrations of the Workers’ Party (PT; *Partido dos Trabalhadores*) (1 January 2001–31 December 2012) and the Brazilian Socialist Party (PSB; *Partido Socialista Brasileiro*) (1 January 2013–present). In this period, as a norm, substitute housing followed eviction. However, not enough houses were built for all evicted residents.
